# An improved system for estradiol-dependent regulation of gene expression in yeast

**DOI:** 10.1186/1475-2859-6-10

**Published:** 2007-03-20

**Authors:** María J Quintero, Douglas Maya, Miguel Arévalo-Rodríguez, Ángel Cebolla, Sebastián Chávez

**Affiliations:** 1 Departamento de Genética, Universidad de Sevilla, Avda. Reina Mercedes 6, E41012-Seville, Spain; 2 Biomedal, SL, E41092-Seville, Spain

## Abstract

**Background:**

*Saccharomyces cerevisiae *is widely utilized in basic research as a model eukaryotic organism and in biotechnology as a host for heterologous protein production. Both activities demand the use of highly regulated systems, able to provide accurate control of gene expression in functional analysis, and timely recombinant protein synthesis during fermentative production. The tightly regulated *GAL1-10 *promoter is commonly used. However, induction of the *GAL *system requires the presence of the rather expensive inducer galactose and the absence of glucose in the culture media. An alternative to regulate transcription driven by *GAL *promoters, free of general metabolic changes, is the incorporation of the hybrid Gal4-ER-VP16 protein developed by D. Picard. This chimeric protein provides galactose-independent activation of transcription from *GAL *promoters in response to β-estradiol, even in the presence of glucose. However, constitutive expression of this transactivator results in relatively high basal activity of the *GAL *promoters, therefore limiting the gene expression capacity that is required for a number of applications.

**Results:**

In order to improve this expression tool, we have introduced additional regulatory elements allowing a simultaneous control of both the abundance and the intrinsic activity of the Gal4-ER-VP16 chimeric transactivator. The most efficient combination was obtained by placing the coding sequence of the hybrid activator under the control of the *GAL1 *promoter. This configuration results in an amplification feedback loop that is triggered by the hormone, and ultimately leads to the enhanced regulation of recombinant genes when these are also driven by a *GAL1 *promoter. The basal expression level of this system is as low as that of native *GAL*-driven genes in glucose-containing media.

**Conclusion:**

The feedback regulatory loop that we have engineered allows a 250-fold induction of the regulated gene, without increasing the basal activity of the target promoter, and achieving a 12-fold higher regulation efficiency than the previous configuration.

## Background

Recombinant DNA expression constitutes a major approach in gene function studies that naturally complement genetic and genomic research. Well-regulated expression systems provide an invaluable tool to investigate the cellular roles of novel genes, either in their original cellular environment, or in specialized host organisms. Thus, these systems can be utilized to observe the biological effects of the controlled expression (or lack of it) of a given DNA sequence. Very often they also provide the means to produce and purify a desired gene product, opening the way to the comprehensive analysis and manufacture of proteins of biotechnological interest.

*S. cerevisiae *has been widely employed as a host organism in the expression of heterologous proteins [1-7], using regulated systems developed to allow low basal, highly inducible protein production. In this sense, timely expression during fermentation is important to prevent a premature metabolic burden and any possible toxic effect throughout the culture growing-phase that might lead to a reduction in protein yields, or to genetic instability. Yeast-derived transcriptional promoters used in the above mentioned systems are, along with others, those of the *MET3 *gene, negatively regulated by the amino acid methionine [8], the *PHO5 *gene, negatively regulated by inorganic phosphate [9], the *CUP1 *gene, activated by Cu^2+ ^ions [10], and the *GAL1 *and *GAL10 *genes, activated by galactose and repressed by glucose [11,12]. Other yeast systems designed for tightly regulated gene expression incorporate transcriptional elements derived from bacteria, like those inducible or repressible by tetracycline (Tet-Off and Tet-On) [13,14].

Expression systems based on the *GAL1-10 *promoter are among the strongest ones [15]. Under natural conditions, expression of the *GAL1 *and *GAL10 *genes depends on the product of the *GAL4 *gene, which activates the *GAL1-10 *promoter in the presence of galactose and the absence of glucose [16], a major disadvantage when the metabolic changes associated to this switch in carbon source are relevant to the study. In addition, the high cost of the inducer can preclude scaling up production of a commercially valuable protein using this system. A good alternative to regulate transcription driven by *GAL *promoters is the incorporation of the hybrid protein developed by D. Picard and co-workers, a chimerical transcriptional activator that combines the DNA binding domain of Gal4 with the hormone binding domain of human estrogen receptor and the transactivation domain of the herpex virus protein VP16 [17]. This system permits the β-estradiol-inducible expression of recombinant genes placed under the control of *GAL *promoters, in a manner that is independent of the presence or absence of galactose or glucose. The system is virtually independent of media formulation and avoids undesired physiological changes associated with carbon source switch, but the constitutive expression of the chimeric transactivator produces increased basal activities of the target promoters. The reported effects of the transactivator on the basal activity of GAL promoters range between 2.3- [18] and 11.4-fold [17]. In this report we describe an improved yeast expression platform based on the system described by Louvion *et al *[17]. This improvement relies on linked regulatory cascades or positive feedback loops that amplify the activating signal of the inducer molecule. Both the cascade and the loop modulate simultaneously the abundance of the transactivator and its intrinsic activity, resulting in low-basal, highly inducible gene expression systems.

## Results and Discussion

### A native *GAL *promoter does not eliminate the basal increase produced by Gal4-ER-VP16

**Figure 1:**
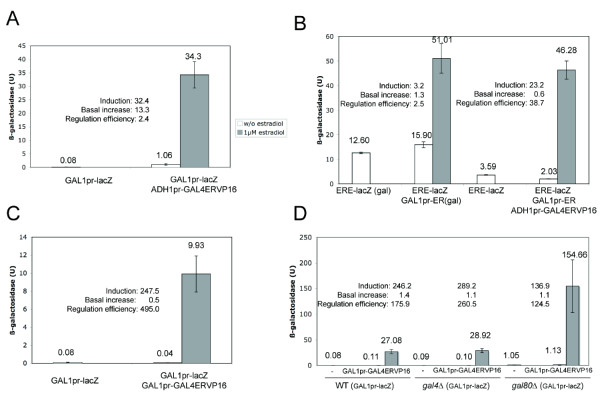
**Hormone-dependent regulation of β-galactosidase by the different systems analysed in this work**. β-galactosidase activities (Miller units) of W303-1A (A, B, C) or BY4741 (D) cells containing the following plasmids: (A) p416GAL1-lacZ and pHCA/GAL4(1-93)ERVP16; (B) pVitBx2 and pGAL1-ER; or pVitBx2, pGAL1-ER and pHCA/GAL4(1-93)ERVP16; (C) p416GAL1-lacZ and p414GAL1-GAL4ERVP16; (D) p416GAL1-lacZ and p415GAL1-GAL4ERVP16. All cultures were grown in the presence of glucose except when "gal" is written, indicating galactose-containing cultures. In all cases, 5 ml cultures were inoculated from a mid-log preculture to an O.D. (600 nm) of 0.02, and incubated for 14 hours at 30°C in the presence or the absence of 1 μM β-estradiol. Average and standard deviation of at least three independent experiments are represented. Induction represents the ratio between β-galactosidase activities (in the presence and in the absence of β-estradiol). Basal increase was calculated dividing the β-galactosidase activity detected in the absence of hormone, by the activity exhibited by cells cultured in the same conditions, containing the same target reporter construct, but lacking any estrogen-dependent transactivator. Regulation efficiency was calculated dividing induction by basal increase.

In order to explore possible ways to improve the efficiency of the yeast estrogen-regulated system described by Louvion *et al *[17], we first tested if the high basal activity levels reported by these authors were due to the use of artificial *GAL *promoters, combining a number of Gal UAS fused to the *CYC1 *TATA sequences. To this end, we measured the effect of a constitutive Gal4-ER-VP16 transactivator on the expression of a reporter gene (*lacZ*), placed under the control of a native *GAL1 *promoter, in the absence of β-estradiol. We found that the constitutive Gal4-ER-VP16 [17] produced a 13.3-fold increase in the activity of the native *GAL1 *promoter (Fig 1A). Addition of β-estradiol to the culture medium produced a further 32.4-fold increase in the activity of the native *GAL1 *promoter (Fig 1A), this induction being weaker than that reported by Louvion *et al. *for the artificial *GAL *promoters (between 100 and 200-fold) [17]. Therefore, a native *GAL *promoter would not improve the regulation capacity of an expression system based on the Gal4-ER-VP16 protein.

### An estradiol-induced transcriptional cascade

**Figure 2:**
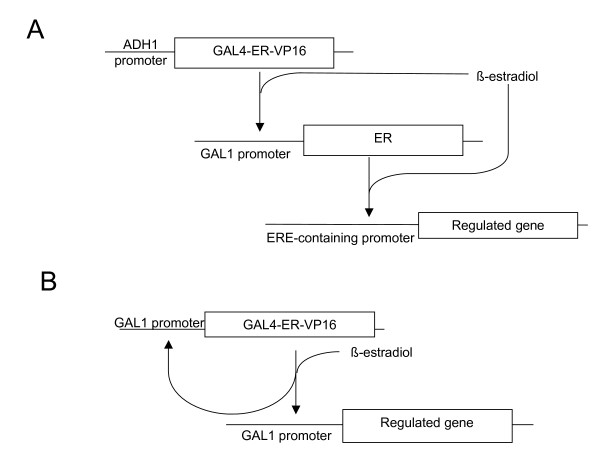
**Synoptic explanation of the two estrogen-induced regulatory systems described in this work**. A. β-estradiol-induced transcriptional cascade. B. β-estradiol-triggered self-induced regulatory loop. Boxes indicate coding regions; arrows indicate activation.

**Figure 3:**
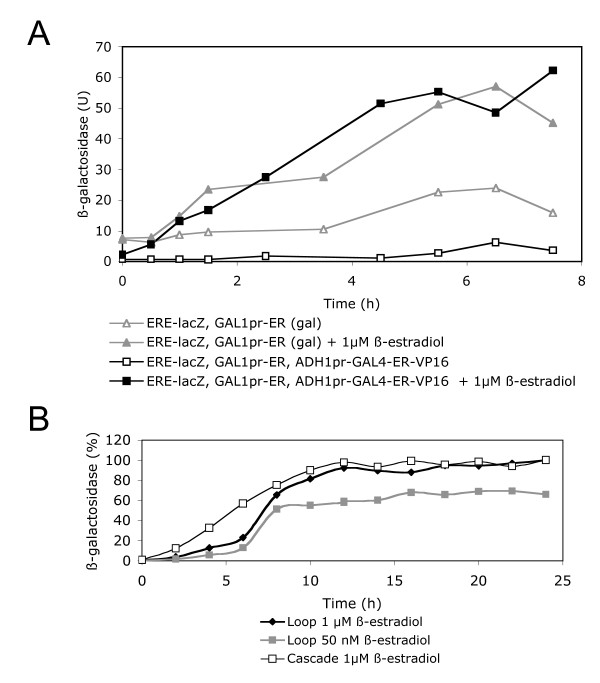
**Induction time-course of the estrogen-dependent transcriptional cascade and of the estradiol-triggered regulatory loop**. A. 25 ml glucose selective medium were inoculated with W303-1A yeast cells, containing plasmids pVitBx2, pHCA/GAL4(1-93)ERVP16 and pGAL1-ER. A similar volume of galactose selective medium was inoculated with yeast cells containing plasmids pVitBx2 and pGAL1-ER. After adding 1 μM β-estradiol (solved in ethanol), or similar amounts of ethanol, samples were taken at the indicated time points and assayed for β-galactosidase (Miller units). A representative experiment is shown. B. 24 hours time-course of W303-1A yeast cells, containing the cascade system (plasmids pVitBx2, pHCA/GAL4(1-93)ERVP16 and pGAL1-ER) after adding 1 μM β-estradiol, compared with W303-1A yeast cells containing the loop system (plasmids p416GAL1-lacZ and p414GAL1-GAL4ERVP16) after adding two different hormone conentration (1 μM and 50 nM). The experiments were performed as in A. To facilitate comparison, the results were represented as the percentage of the maximal values reached by each culture, except for the loop 50 nM estradiol, wich was referred to the maximal value of the loop culture containing 1 μM estradiol. A representative experiment is shown.

We investigated the possibility of linking the Gal4-ER-VP16-regulated system upstream to a second, β-estradiol-dependent expression system. As this second system we chose a *GAL1 *promoter-driven human estrogen receptor (ER) and a target reporter for this transactivator (ERE-lacZ), consisting of a minimal *CYC1 *promoter fused to three estrogen-responsive elements (ERE) and driving *lacZ *transcription. The combination of the *GAL1*pr-controlled ER with the constitutively expressed Gal4-ER-VP16 would produce a transcriptional cascade that should be active only in the presence of hormone. Its final activator (ER) is controlled by β-estradiol, at both its expression level and its transactivator activity (Fig 2A). As shown in Fig. 1B, in the presence of galactose the *GAL1p*r-ER/ERE-*lacZ *system alone showed very low activation when 1 μM β-estradiol was added (3.2-fold induction). This is a poor induction value, in comparison to previous studies of ER-dependent regulation in yeast [19-22], probably due to the high intrinsic activity of the ERE-lacZ reporter. However, combination of the *GAL1p*r-ER/ERE-*lacZ *system with the constitutively expressed Gal4-ER-VP16 in the "cascade-like" configuration resulted in a β-estradiol-inducible system with a reduced basal increase and an improved (23.2-fold) induction level. The induction time courses of the combined *ADH1*pr-Gal4-ER-VP16/*GAL1p*r-ER/ERE-*lacZ *system in glucose plus β-estradiol and the *GAL1p*r-ER/ERE-*lacZ *in galactose were similar (Fig 3A), indicating that the higher complexity of the cascade system does not involve a slower response. The cascade induction reached a plateau 10–12 hours after the addition of the hormone (Fig 3B).

The global efficiency of a regulated expression system depends on both the induction level and the increase in basal activity of the target promoter due to the presence of its associated activator. In order to compare the different systems tested we introduced a new parameter, called *regulation efficiency*, obtained as the ratio between the induction level and the increase in basal activity observed with each system. Thus, the system composed by the constitutively expressed *ADH1*pr-Gal4-ER-VP16 transactivator and the *GAL1 *promoter showed a regulation efficiency of 2.4. A similarly low value was exhibited by the *GAL1p*r-ER/ERE-*lacZ *system for β-estradiol in the presence of galactose. In contrast, the *ADH1*pr-Gal4-ER-VP16/*GAL1p*r-ER/ERE-*lacZ *system showed a regulation efficiency of 38.7, indicating that the sequential action of two regulators is more convenient when an accurate regulation is needed. This improved regulation resulted from the combination of a higher induction level (23.2-fold) with a slight reduction of the basal activity of the ERE-lacZ reporter (0.6-fold). However, according to the values reported by Louvion *et al*., the regulation efficiency of their system ranged between 11.0 and 40.5 [17]. We therefore conclude that the transcriptional cascade assembled in these studies, although resulting in a moderately low basal activity of the target promoter, does not significantly improve the regulation efficiency of the original Gal4-ER-VP16-dependent system.

### An estradiol-triggered self-induced regulatory loop

As an alternative approach, and in order to prevent the increase in basal activity of *GAL *promoters produced by the constitutive expression of Gal4-ER-VP16, we placed the transcription of this chimeric transactivator under the control of a *GAL1 *promoter. The resulting system, designed as a self-inducible regulatory loop triggered by β-estradiol is depicted in Fig 2B. First, we measured the expression of a *GAL1pr*-*lacZ *reporter construct in cells containing this regulatory loop in the absence of estrogen, finding no increase in the basal activity of the *GAL1 *promoter, but rather a small decrease (Fig 1C). When these cells were cultured in the presence of 1 μM β-estradiol, we measured a 247.5-fold induction of *lacZ *expression. Although the overall maximal activity was 3.5-fold higher when the activator was constitutively expressed (Fig 1A,C), the induction of the loop exceeded the highest induction level reported by Louvion *et al. *[17] and it was 7 times higher than that produced by the constitutively expressed Gal4-ER-VP16 system on the same target promoter (Fig 1A).

Considering altogether the small reduction in basal activity and the considerable induction level observed, the resulting regulation efficiency of the self-inducing system was very high (495.0). Although the basal activities of the loop system are close to zero, and therefore this number should be considered cautiously, it represents one order of magnitude higher than the values found for the constitutively expressed Gal4-ER-VP16 system or the transcriptional cascade described above. The tighter regulation of this loop system involved an exponential kinetic of induction rather than linear kinetic shown by the cascade system, as indicated by the induction time-courses shown in Fig 3B. However, both the cascade and the loop systems reached the maximal induction 10–12 hours after adding the hormone (Fig 3B).

The high efficiency of this regulatory loop is not restricted to the W303 yeast genetic background. When we introduced the GAL1pr-GAL4ERVP16/GAL1pr-lacZ system in an S288C-derivative strain (BY4741), similar induction levels (246-fold) and regulation efficiency (176) were obtained (Fig 1D). Moreover, in the S288C background the maximal activities were higher than in W303 (Fig 1C,D).

In order to characterize the loop system further, we investigated its dependence on Gal4 and Gal80, the two specific regulators of the *GAL1 *promoter. As shown in Fig 1D, the absence of the Gal4 activator did not affect significantly either the basal or the induced levels. In the absence of the Gal80 repressor, the basal activity of the *GAL1 *promoter was increased, as could be expected. Again the loop system caused a significant induction in this background (Fig 1D). These results suggest that the loop system can be easily adapted to other eukaryotic cells lacking the Gal4 and Gal80 regulators.

**Figure 4:**
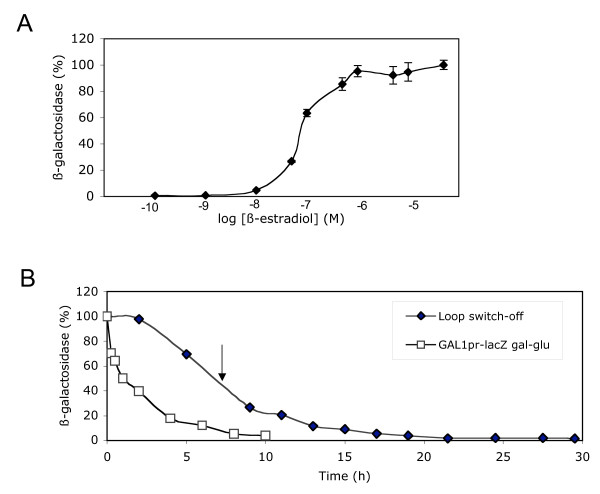
**Hormone-dependence and switch-off time course of the β-estradiol-triggered self-induced regulatory loop**. 5 ml glucose cultures of W303-1A cells containing plasmids p416GAL1-lacZ and p414GAL1-GAL4ERVP16 were inoculated as in Fig 1 and grown for 14 hours in the presence of the indicated concentration of β-estradiol and assayed for β-galactosidase. The average and standard deviation of three different experiments is represented. B. 100 ml glucose medium were inoculated with yeast cells containing plasmids p416GAL1-lacZ and p414GAL1-GAL4ERVP16 and grown until stationary phase (19 h) in the presence of 50 nM estradiol. Cells were then washed three times by centrifugation and used to inoculate a new 100 ml culture lacking β-estradiol, at a OD(600 nm) of 0.2. Seven hours later (arrow) cells were washed again and used to inoculate a new culture at a OD(600 nm) of 0.2. As a control, a 100 ml glucose culture was inoculated at a OD(600 nm) of 0.2, from a saturated preculture grown in galactose. Samples were taken at the indicated times and assayed for β-galactosidase activity. A representative experiment is shown

We also measured the hormone dose-response of the loop system. The levels obtained after culturing the yeast cells with increasing concentrations of estradiol are shown in Fig 4A. Maximal induction was observed at about 1 μM and the half-maximal response at 0.05 μM. These values are one order of magnitude higher than those reported for the constitutively expressed Gal4-ER-VP16 system [17] and for the hormone-dependence of ER in yeast [19,21]. In fact, the dose response of the loop system does not show the typical sharp increase of hormone receptors, suggesting that the self-induced loop facilitates to obtain intermediate levels of expression. Moreover, the time course of the induction at sub-optimal hormone concentration (50 nM) also indicated an exponential kinetic, but at this ligand concentration the loop system reached the plateau 2–4 hours earlier than the culture activated with 1 μM hormone (Fig 3B).

We also tested if the active loop could be switched-off by removing β-estradiol from the medium. As shown in Fig 4B, five hours and two cell duplications after removing the hormone, cells retained nearly 70% of the β-galacosidase activity. A new reinoculation and eight more hours were needed to reduce the β-galactosidase activity below 10% of the initial values. The comparison with the switch-off of a galactose-activated *GAL1pr*::lacZ, by transferring the cells from galactose to glucose, rules-out that the slow inactivation of the loop might be due to the high stability of β-galactosidase (Fig 4B). One possible explanation for these results is that the residual amounts of estradiol remaining inside the cell were sufficient to maintain the activation of the system. An alternative explanation might be that, once the regulatory loop has been triggered, the system remains epigenetically active without needing additional inputs. In any case, this characteristic of the system makes it suitable for regulating gene expression in those industrial applications requiring the absence of β-estradiol in the final product. In this study we have tested two new regulation strategies: a transcriptional cascade and a feed back regulatory loop. In both cases we have obtained very low levels of increase in basal activity and considerable levels of induction, especially in the feed back loop. Both systems share a basic feature: the inducer acts on the transactivator simultaneously at two levels: its intrinsic activity and its abundance. We believe that this scheme can be used to improve the regulation capacity of other expression systems, whose regulators produce high basal activity of the target promoters.

## Conclusion

The coordinated control of the expression of an estrogen-responsive transcriptional activator with its intrinsic activation amplifies synergistically the regulatory capacity of the target promoter.

The transcriptional cascade described in this work, composed by a constitutively expressed Gal4-ER-VP16 transactivator and a *GAL1*pr-driven estrogen receptor, does not increase the basal activity of the ERE-containing target promoter, maintaining the regulation efficiency of the previous system.

The self-induced regulatory loop that we have engineered allows a 250-fold induction of the regulated gene, without increasing the basal activity of the target promoter. The global regulation efficiency of this feed back loop exceeds the estrogen-induced expression systems that have been previously designed for yeast cells. Its hormone-dependence, more gradual than the one usually exhibited by nuclear receptors, allows intermediate levels of regulation. The loop system does not depend on the specific regulators of the *GAL1 *promoter, facilitating its adaptation to other eukaryotic environments.

## Methods

### Strain and plasmids

The yeast strains used in this study were W303-1A (***MAT*a ***ade2-1 can1-100 his3-11, 15 leu2-3,112 trp1-1 ura3-1*) [23] BY4741 (***MAT*a ***his3Δ1 leu2Δ0 met15Δ0 ura3Δ0*) [24] and the *gal4Δ *and *gal80Δ *derivative of BY4741 conserved in the EUROSCARF collection.

All plasmids used in this study were centromeric and are described in Table 1. Plasmid pGAL1-ER was constructed by inserting a 2.1 kb BamHI DNA fragment from plasmid pP6HEG0 [25], containing the cDNA of human estrogen receptor, into the BamHI site present in p414GAL1 [26], immediately downstream from the *GAL1 *promoter. Plasmid p414GAL1-GAL4ERVP16 was constructed by inserting a 1,5 kb PCR fragment (oligos ATGAAGCTACTGTCTTCTATCGA and TCACTATAGGGCGAATTGG) from pHCA/GAL4(1-93)ERVP16, encoding the chimeric transactivator GAL4ERVP16 [17], into pGEMT-easy (Promega), generating pGEMT-GAL4ERVP16. A 1.5 kb SpeI fragment from this plasmid was inserted into the SpeI site present in p414GAL1 [26], immediately downstream from the *GAL1 *promoter. Plasmid p415GAL1-GAL4ERVP16 was constructed by ligating the 2.6 kb PvuII fragment from p414GAL1-GAL4ERVP16 to the bigest PvuII fragment of pRS415 [27].

**Table 1:** Plasmids used in this work

Plasmid	Relevant features	Reference
p416GAL1-lacZ	*URA3*, *CEN*, *GAL1*pr::*lacZ*	[26]
pHCA/GAL4(1-93)ERVP16	*HIS3*, *CEN*, *ADH1pr::GAL4ERVP16*	[17]
pVitBx2	*URA3*, *CEN*, ERE*-CYC1*pr::*lacZ*	[30]
pGAL1-ER	*LEU2*, *CEN*, *GAL1pr::ER*	This study
p414GAL1-GAL4ERVP16	*TRP1*, *CEN*,*GAL1pr::GAL4ERVP16*	This study
p415GAL1-GAL4ERVP16	*LEU2*, *CEN*,*GAL1pr::GAL4ERVP16*	This study
pP6HEG0	human ER	[25]
pRS415	*LEU2*, *CEN*	[27]
p414GAL1	*TRP1*, *CEN*, *GAL1pr*	[26]

Yeast transformation was carried out using standard methods [28]. Transformants were routinely assayed for plasmid stability, by replica-plating after non-selective culturing. Those transformants containing more than one plasmid did not show abnormal levels of plasmid instability.

### Culture conditions

Yeast transformants were cultured at 30°C in complete synthetic medium, with 2% glucose or 2% galactose as the carbon source [28]. Liquid cultures were incubated in orbital shakers at 120 rpm. β-estradiol (SIGMA E-1024-1G) was added when indicated from a 2.5 mM stock solution in ethanol. Equivalent amounts of ethanol were added to control cultures.

### β-galactosidase assays

β-galactosidase assays were performed as described previously [29] using a cell-permeabilization method [28]. Activities were calculated as Miller units. Yeast strains containing reporters alone were assayed in the presence of empty expression vectors. Experiments were performed at least three times, although in figures representing time-course experiments only one is shown. In all cases the replicate produced similar results.

## Competing interests

The University of Seville is currently applying for a patent covering the applications of the results described in this work. Biomedal, SL provided a part of the funding required to develop this work and has signed a licence agreement with the University of Seville, in order to bring to the market some applications of these results.

## Authors' contributions

MJQ carried out most of the experiments and contributed to their design. DM performed some experiments. MAR participated in the design of some experiments and contributed to draft the manuscript. AC contributed to the design of some experiments and, together with SC, conceived the initial approaches. SC performed some experiments, coordinated all the work and drafted the manuscript.
